# Data on the expression of PEPCK in HepG2 hepatocytes transfected with miR-195

**DOI:** 10.1016/j.dib.2017.10.061

**Published:** 2017-10-28

**Authors:** Won-Mo Yang, Kyung-Ho Min, Se-Whan Park, Wan Lee

**Affiliations:** a Department of Biochemistry, Dongguk University College of Medicine, Gyeongju-si, Gyeongsangbuk-do 38067, Republic of Korea; b Endocrine Channelopathy, Channelopathy Research Center, Dongguk University College of Medicine, Goyang-si, Gyeonggi-do 10326, Republic of Korea

**Keywords:** MicroRNAs, miR-195, PEPCK, Obesity, Hepatocyte

## Abstract

Dietary fats rich in saturated fatty acid (SFA) increase the risk of metabolic diseases, and certain microRNAs (miRNAs) dysregulated by SFA are associated with the pathogenesis of insulin resistance and type 2 diabetes. A previous study found that miR-195 is increased by SFA and impairs hepatic insulin signaling through the suppression of INSR (Yang et al., 2014) [1]. This article reports accompanying data to determine the effect of miR-195 on the expression of PEPCK, a key player in hepatic gluconeogenesis. The transfection of miR-195 in HepG2 hepatocytes was found to increase the mRNA and protein expression of PEPCK. Moreover, the insulin-stimulated reduction of PEPCK expression was attenuated drastically by miR-195. More detailed analysis and understanding of the role of miR-195 in diet-induced hepatic insulin resistance can be found in "Saturated fatty acid-induced miR-195 impairs insulin signaling and glycogen metabolism in HepG2 cells" (Yang et al., 2014) [1].

**Specifications Table**

**Table:** 

Subject area	*Cell biology*
More specific subject area	*MicroRNA, Metabolism, Obesity*
Type of data	*Figures and text*
How data was acquired	*Analysis of RT-PCR, qRT-PCR, and immunoblotting*
Data format	*Analyzed*
Experimental factors	*Transfection of miR-195, Treatment of insulin, Analysis of the mRNA and protein expression of PEPCK*
Experimental features	*HepG2 cells were transfected with scRNA or miR-195 mimic. For insulin stimulation, 100 *nM *of insulin was treated during the last 30 *min *of incubation.*
Data source location	*Dongguk University School of Medicine, Gyeongju-si, Gyeongsangbuk-do 38067, Korea*
Data accessibility	*The data are available with this article*

**Value of the data**

• The data can be compared with diverse target genes of miR-195 in different cell type.
• The data highlight the biological significance of miR-195 in PEPCK expression and hepatic insulin resistance.
• The data are useful in understanding the regulatory mechanism of hepatic gluconeogenesis and insulin sensitivity modulated by miR-195.

## 1 Data

**Fig. 1:**
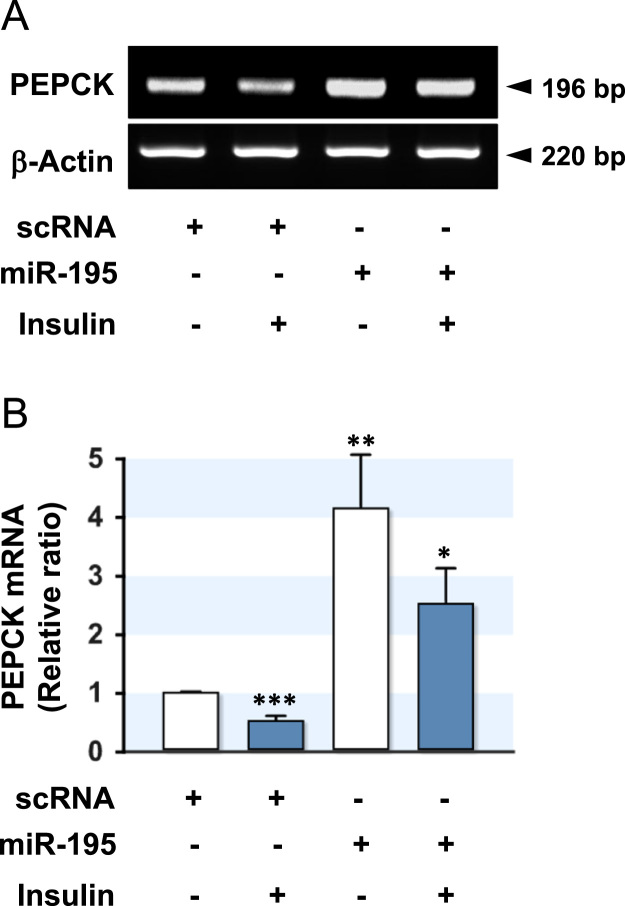
Effect of miR-195 on the mRNA expression of PEPCK. HepG2 cells were reverse-transfected with scRNA (200 nM) or miR-195 (200 nM). The mRNA level of PEPCK was analyzed at 48 h after reverse-transfection by both RT-PCR (A) and *q*RT-PCR (B). The values are expressed as the relative ratio, where the intensity of the basal scRNA control was set to one. Values are means ± SEM. *, *P* < 0.05; **, *P* < 0.01; ***, *P* < 0.001 vs. basal scRNA control.

**Fig. 2:**
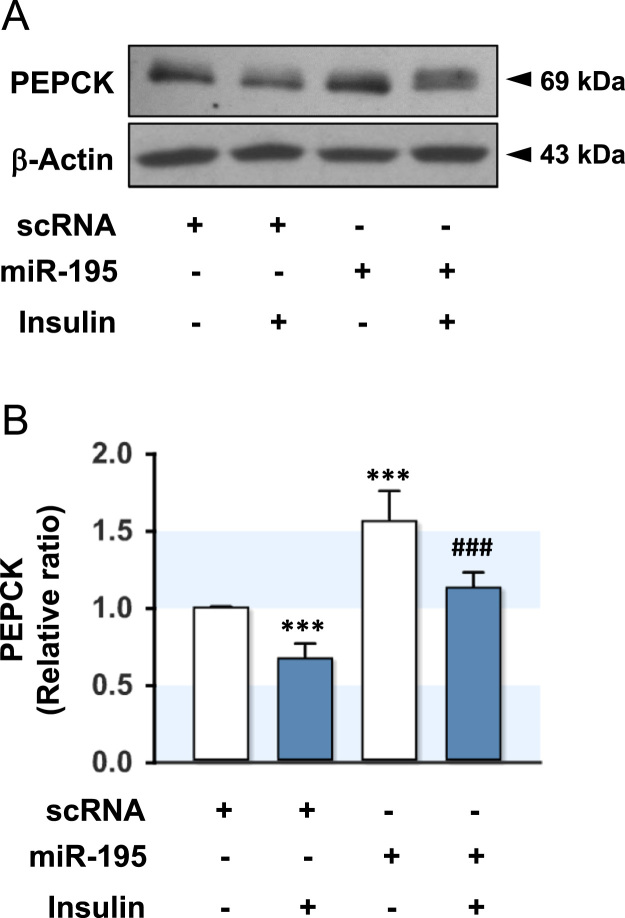
Effect of miR-195 on the protein expression of PEPCK. HepG2 cells were reverse-transfected with scRNA (200 nM) or miR-195 (200 nM). After 48 h transfection, the protein expression of PEPCK was analyzed by immunoblotting. (A) Representative immunoblot obtained from three independent experiments were shown. (B) The immunoblot intensities for PEPCK/β-Actin were quantified by densitometry and expressed in relative ratio. The intensity of basal scRNA control was set to one. Values are expressed as means ± SEM of three independent experiments. ***, *P* < 0.001 vs. basal scRNA control; ###, *p* < 0.001 scRNA + Insulin vs. miR-195 + Insulin.

The excess accumulation of lipids in the liver is a major causal factor in the pathogenesis of insulin resistance, type 2 diabetes, and metabolic syndrome [2]. The dysregulation of certain miRNAs derived from diet-induced obesity has been implicated in the expression regulation of insulin signaling molecules and the pathogenesis of insulin resistance [3], [4]. Previously, miR-195 was reported to be upregulated by a diet high in saturated fatty acids and impairs hepatic insulin signaling through the suppression of INSR [1]. Therefore, a dysregulation of mir-195 expression is linked to the development of hepatic insulin resistance and type 2 diabetes. This article reports accompanying data to determine further the effects of miR-195 on the expression of PEPCK, a key player in hepatic gluconeogenesis. HepG2 hepatocytes were transfected with the scRNA control or miR-195 mimic, and the mRNA (Fig. 1A and B) and protein (Fig. 2A and B) expression of PEPCK were analyzed either in the presence or absence of insulin. In the scRNA control, insulin reduced the levels of mRNA and protein of PEPCK compared to the basal state. On the other hand, transfection of the miR-195 mimic in hepatocytes increased the mRNA and protein levels of PEPCK significantly. Moreover, the insulin-stimulated reduction of PEPCK expression was attenuated drastically by miR-195 transfection. Thus, an increase in miR-195 lead to upregulation of PEPCK in hepatocytes. Further analysis of the data and discussion for the implication of miR-195 in hepatic insulin signaling, glycogen synthesis, and the pathogenesis of type 2 diabetes are presented in "Saturated fatty acid-induced miR-195 impairs insulin signaling and glycogen metabolism in HepG2 cells" [1].

## 2 Experimental design, materials and methods

### 2.1 Cells and culture condition

HepG2, a human liver cancer cell line, was obtained from ATCC (#77400). The HepG2 cells was cultured in MEMα supplemented with 10% FBS and 1% penicillin-streptomycin (Gibco) in an atmosphere containing 5% CO_2_ at 37 °C. The cells from passages 4 to 10 were used in subsequent experiments.

### 2.2 Transfection of miRNA mimics

The miRNA mimics and scRNA were purchased from Genolution (Seoul, Korea). HepG2 cells were transfected with the 200 nM mimics of scrambled control miRNA (scRNA) or miR-195 using G-fectin (Genolution) according to the manufacturer's instructions. After 48 h transfection, expression of PEPCK was analyzed by RT-PCR, *q*RT-PCR, and immunoblotting.

### 2.3 RNA extraction and analysis of PEPCK mRNA

The total RNA from the HepG2 cells was extracted using a miRNeasy Mini Kit (Qiagen) according to the manufacturer's instructions. The purity and integrity of the RNA were assessed using a ND-1000 Spectrophotometer (NanoDrop) and Agilent 2100 Bioanalyzer (Agilent Technologies). A 2 μg of RNA from was reverse transcribed and the cDNA obtained was used to analyze the PEPCK transcript levels by RT-PCR and *q*RT-PCR with the forward primer: 5′-CAA TGC CGA CCT CCC CTG TG-3′ and reverse primer: 5′-CTG CTC CCG GTG TGG TGA TG-3′. RT-PCR and *q*RT-PCR were conducted using the GoTaq Green Master Mix (Promega) and SYBR Green PCR Master Mix (Applied Biosystems), respectively. The data of *q*RT-PCR were analyzed by the advanced relative quantification method in Light-Cycler 480 software (Roche Diagnostics). β-Actin were applied as the internal control on the expression levels of the mRNAs.

### 2.4 Cell lysis, immunoblotting, and antibodies

The cells were lysed using a lysis buffer and Laemmli solution. SDS-gel electrophoresis and immunoblotting analysis were conducted, as described elsewhere [5]. The antibody against PEPCK was purchased from Abcam (Cambridge, UK). Anti-β-actin antibody was obtained from Santa Cruz Biotechnology (Santa Cruz, CA, US). The proteins were visualized using the ECL Western Blotting Detection Reagent (GE Healthcare, Buckinghamshire, UK). The immunoblot intensities were quantified by densitometry using an analytical scanning system (Alpha Imager HP; Alpha Innotech, San Leandro, CA, US).

### 2.5 Database and statistical analysis

The experimental values are expressed as the means ± SEM from three independent experiments. Where applicable, the significance of the difference was analyzed using a Student's *t*-test for unpaired data.
